# Development of an AI-based magnetic resonance imaging reading support program (AMP) for deep endometriosis diagnosis

**DOI:** 10.1038/s41598-025-30277-x

**Published:** 2025-12-08

**Authors:** Rie Shiokawa, Junichiro Iwasawa, Yumiko Oishi Tanaka, Yuta Tokuoka, Yohei Sugawara, Yuichiro Hirano, Ryo Takaji, Yayoi Hayakawa, Keita Oda, Yasunori Kudo, Miho Li, Kazue Mizuno, Kazuhisa Ozeki, Ayako Nishimoto-Kakiuchi, Kimio Terao

**Affiliations:** 1 Chugai Pharmaceutical Co., Ltd., 1-1 Nihonbashi-Muromachi 2-chome, Nihonbashi Mitsui Tower (Reception12F), Chuo-ku, Tokyo, 103-8324 Japan; 2 Preferred Networks, Inc., Tokyo, 100-0004 Japan; 3 Diagnostic Imaging Center, Cancer Institute Hospital of Japanese Foundation for Cancer Research, Tokyo, 135-8550 Japan; 4 Department of Radiology, IUHW Narita Hospital, Chiba, 286-8520 Japan; 5 Department of Radiology, Oita University Faculty of Medicine, Oita, 879- 5593 Japan; 6 Department of Radiology, Juntendo University School of Medicine, Tokyo, 113-8421 Japan

**Keywords:** Deep endometriosis, Diagnosis, MRI, Artificial intelligence, Machine learning, Radiologist, Biomarkers, Computational biology and bioinformatics, Diseases, Medical research

## Abstract

Diagnosis of endometriosis faces significant challenges including diagnostic delay and reliance on invasive procedures. Deep endometriosis (DE) poses additional difficulties in non-invasive diagnosis due to its subtle and complex imaging features. To address these challenges, we developed an AI-based MRI reading support program (AMP) designed to improve diagnostic accuracy and efficiency, with the primary endpoint of demonstrating its potential to enhance radiologists’ reading sensitivity. AMP comprises the following three models: (1) a nnU-Net model for endometriotic nodular lesion (plaque) segmentation, (2) a radiomics-based LightGBM model for adhesion detection, and (3) a nnU-Net model for detection/quantification of ovarian endometriotic cysts (OECs). In cross-validation, AMP achieves mean Dice similarity coefficient of 0.293 for plaque segmentation and 0.580 for OEC segmentation. For adhesion detection, AMP shows high performance for uterine adhesions (F1 scores > 0.6). In a preliminary clinical utility study with three radiologists, AMP improved mean recall for plaque detection from 0.73 to 0.91 demonstrating AMP’s ability to support radiologists in identifying subtle DE lesions and adhesions. Our findings show that AMP is a reliable non-invasive clinical diagnosis tool, that has the potential to minimize diagnostic delays and improve patient outcome.

**Supplementary Information:**

The online version contains supplementary material available at 10.1038/s41598-025-30277-x.

## Introduction

Endometriosis affects approximately 5–10% of women of reproductive age and is associated with chronic pelvic pain and infertility. Up to 50% of affected women experience infertility, with 30% suffering from both pain and infertility^1–5^. Deep endometriosis (DE) is the most aggressive subtype, characterized by invasive nodular lesions that cause adhesions to pelvic organs such as uterosacral ligaments, rectovaginal septum, bladder, and rectum. These lesions frequently cause severe pain and often require surgical intervention.

Definitive diagnosis of endometriosis traditionally relies on invasive procedures such as laparoscopy, which carry a high risk of complications and may lead to irreversible declines in fertility. Moreover, diagnostic delays are a common issue^6–8^. Recently, non-invasive imaging modalities such as transvaginal ultrasonography (TVUS) and magnetic resonance imaging (MRI) have gained attention for early diagnosis and intervention^9,10^. TVUS is highly accurate for detecting ovarian endometriotic cysts (OECs) but has limitations in identifying DE lesions. MRI, with its excellent soft tissue contrast and multiplanar capability, is more suitable for evaluating DE and associated adhesions^11–13^.

However, DE lesions are often small, irregularly shaped, and indistinct from surrounding tissues, making them difficult to detect even for board-certified radiologists^14,15^. The shortage of such radiologists specializing in gynecologic imaging contributes to diagnostic delays and variability in interpretation. Therefore, technological advancements in diagnostic tools and improved efficiency in image interpretation could bring substantial value to endometriosis care.

Artificial intelligence (AI), particularly machine learning (ML), has emerged as a promising approach to enhance diagnostic accuracy and efficiency in medical imaging. While AI applications have progressed in areas such as cancer, cardiovascular and neurological diseases^16–18^, and applied to pelvic MR image analysis primarily focus on ovarian^19^ or endometrial cancer^20,21^, its use in endometriosis—especially DE—remains limited due to small datasets and annotation challenges^22^.

In this study, we developed an AI-based MRI reading support program, AMP, to assist in DE diagnosis. AMP integrates three models: (1) a nnU-Net model^23,24^ for detecting endometriotic nodular lesions (plaques), (2) a LightGBM model^25^ using radiomics features^26^ for adhesion detection, and (3) a nnU-Net model for OEC identification and quantification. The primary endpoint of this study is to develop AMP which supports radiologists’ diagnosis, and to demonstrate its potential to improve sensitivity in reading MR images. To this end, sensitivity improvement was evaluated in a preliminary study, and its utility was further assessed through subjective analysis.

## Materials and methods

### Ethics statement

All studies were conducted in accordance with the Declaration of Helsinki and the International Ethical Guidelines for Biomedical Research involving human subjects. All MR images were obtained from participants who provided written informed consent as part of non-interventional studies approved by the Institutional Review Board at the study institutions (A) Kurashiki Medical Center, (B) Medical Topia Soka Hospital, (C) National University Hospital, (D) Singapore General Hospital, (E) Mayo Clinic Rochester, (F) Mayo Clinic, (G) KK Women’s and Children’s Hospital, (H) Taipei Veterans General Hospital, and (I) Taichung Veterans General Hospital.

### Study population

This retrospective study analyzed a dataset of 333 MR images from 277 patients, with each image comprising one or more tomographic sequences acquired during individual MRI examinations. Among them, 56 patients underwent two MRI examinations—one before and another six months after laparoscopic surgery. Major inclusion criteria are (1) patients aged over 18 years old, (2) a diagnosis of endometriosis was confirmed by gynecologists using diagnostic imaging (TVUS and MRI).

### MR image acquisition and data preprocessing

The MRI protocols used in this study incorporated axial turbo spin-echo T1-weighted imaging (T1WI) and sagittal T2-weighted imaging (T2WI). For sagittal images, the longitudinal planes were set parallel to the main body of the uterus; for axial images, the transverse planes were a mixture of images taken in oblique planes perpendicular to the axis of the corpus and cervix and images taken in planes perpendicular to the axis of the body. In the MR images obtained from institutions (A) and (B), proficient radiologists of an imaging contract research organization (MNES Inc., Hiroshima) performed volume segmentation annotations for the uterus, bladder, rectum, and ovary on the T2WI sequence, and for the OEC on the T1WI sequence. The proficient radiologists also conducted initial volume segmentation annotations for the plaques on the posterior surface of the uterus on T2WI sequences. For MR images from institutions (C)–(I), an early version of the segmentation model provided the preliminary annotations, which were later finalized by Y.O.T who is one of the expert radiologists (more than 15 years’ experience) employing a human-in-the-loop approach^27^. The human–in–the–loop method was essential since distinguishing fibrotic plaques can be challenging even for expert radiologists. All volume segmentation annotations were performed by using the 3D Slicer software^28^. Note that while we employed a 3D convolution-based model that processes the input by stacking multiple 2D sectional images of a sequence into a 3D volume to capture spatial context, it was limited to using a single MR imaging sequence. Although a multi-planar or fully 3D approach that integrates multiple sequences could potentially capture anatomical variability and improve segmentation accuracy, integrating such multiple planes or volumes would require complex data handling and extensive manual annotation by expert radiologists, which was not feasible in this exploratory phase.

All images used in the study were normalized by subtracting the mean value and dividing by the standard deviation of each respective input volume. Furthermore, all images were uniformly resampled to a voxel size of 0.6 $\:\times\:$ 0.6 $\:\times\:$6.0 mm. During the training process, the input images underwent augmentation, which included a random zoom within the range of (0.9, 1.2), a random nonlinear transformation applied to the image’s intensity histogram, an intensity shift facilitated by a randomly chosen offset from (− 0.1, 0.1) of the image standard deviation, and adding Gaussian noise based on a normal distribution of $\mathcal{N}\left( {0,~0.1} \right)$. Following these augmentations, the images were further cropped to a patch size of 384 × 384 × 24 voxels for training. For inference of the entire volume, a sliding window method was employed.

**Fig. 1:**
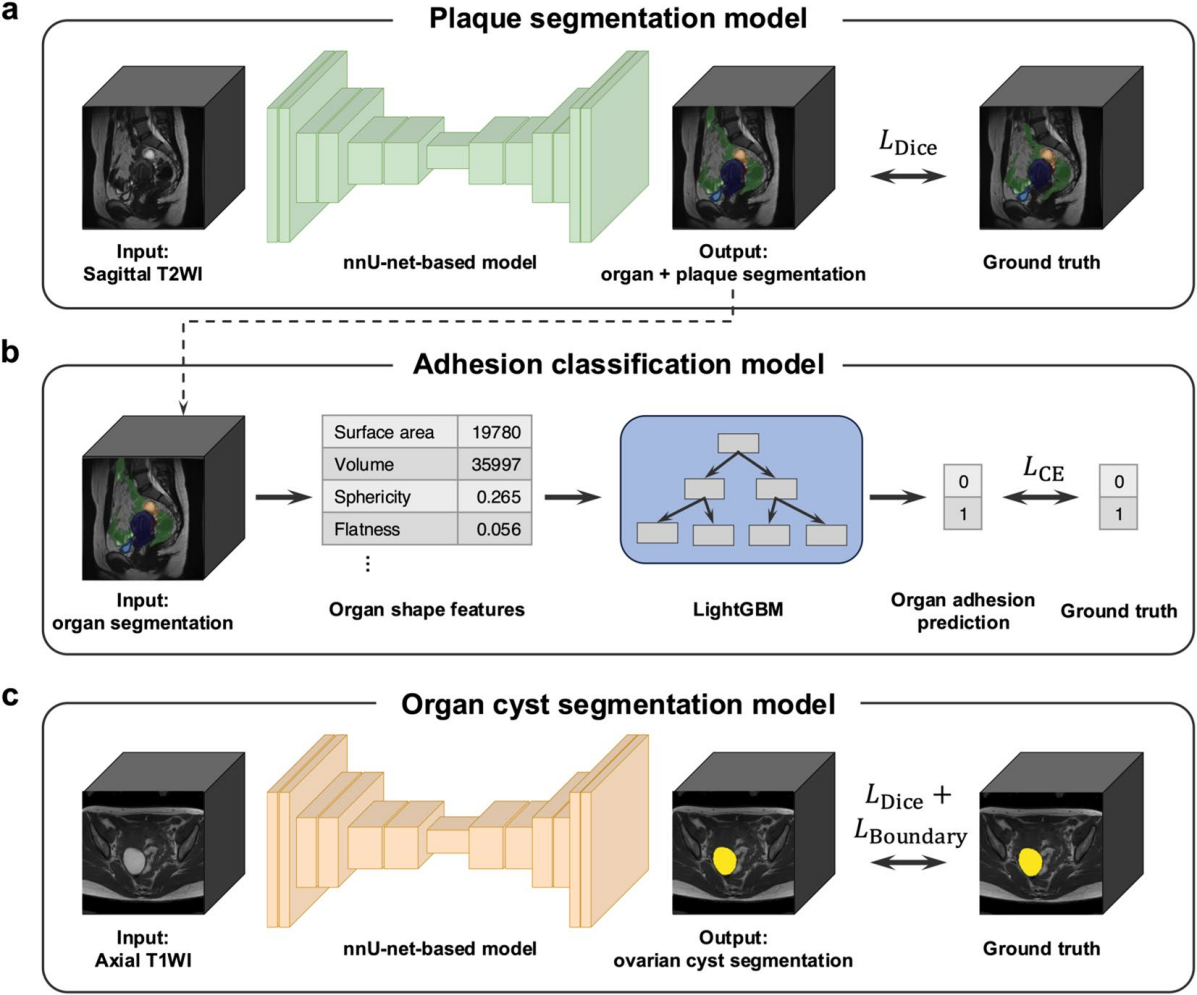
Schematic figure of the suite of models in AMP utilized in this study. a The plaque segmentation model accepts T2WI sagittal sequences as input and returns the segmentation masks for four organs and the plaques. b The adhesion classification model calculates the radiomics shape features of the organ segmentation results obtained by the plaque segmentation model, employs LightGBM to predict the presence of organ adhesions. c The ovarian cyst segmentation model utilizes TIWI axial sequences as input and generates the segmentation mask for ovarian cysts.

**2:**
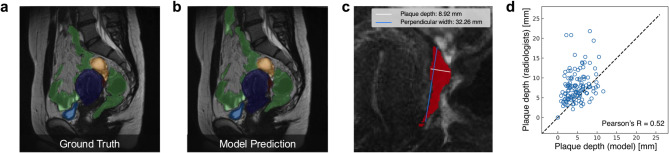
Applying the nnU-Net-based model to evaluate plaque location and thickness. a The ground truth and b model prediction for the segmentation masks of the plaque and four organs which include the uterus (blue), bladder (light blue), rectum (green)*, ovary (yellow) and plaque (red). c The magnified view of b focusing on the segmentation of the plaque (red). The plaque depth measured from the posterior surface of the uterus and its perpendicular width are shown in white and blue, respectively. d The relationship between the automatically measured plaque depth based on the model’s segmentation mask and the plaque depth directly measured from the MR image by an expert radiologist. * In this study, the sigmoid colon was included in the “rectum” as a target area in some cases.

**Fig. 3:**
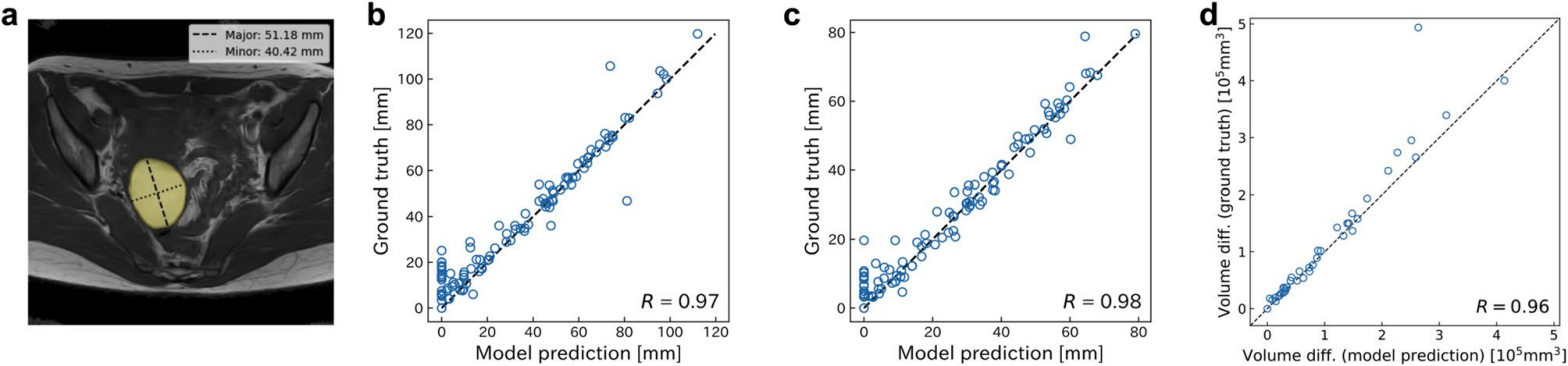
Performance of the OEC model in OEC size evaluation. a An example of the major/minor axis measurement derived from the predicted segmentation mask of the OEC by the model. b, c The relationship between the automatically computed b major or c minor axis length of the OEC based on the model’s segmentation mask and that determined by the expert and proficient radiologists’ annotations. d The relationship between the volume change of the OEC at pre- and post-surgery measured from the model’s predicted segmentation mask and that determined by the expert and proficient radiologists’ annotations.

**Fig. 4:**
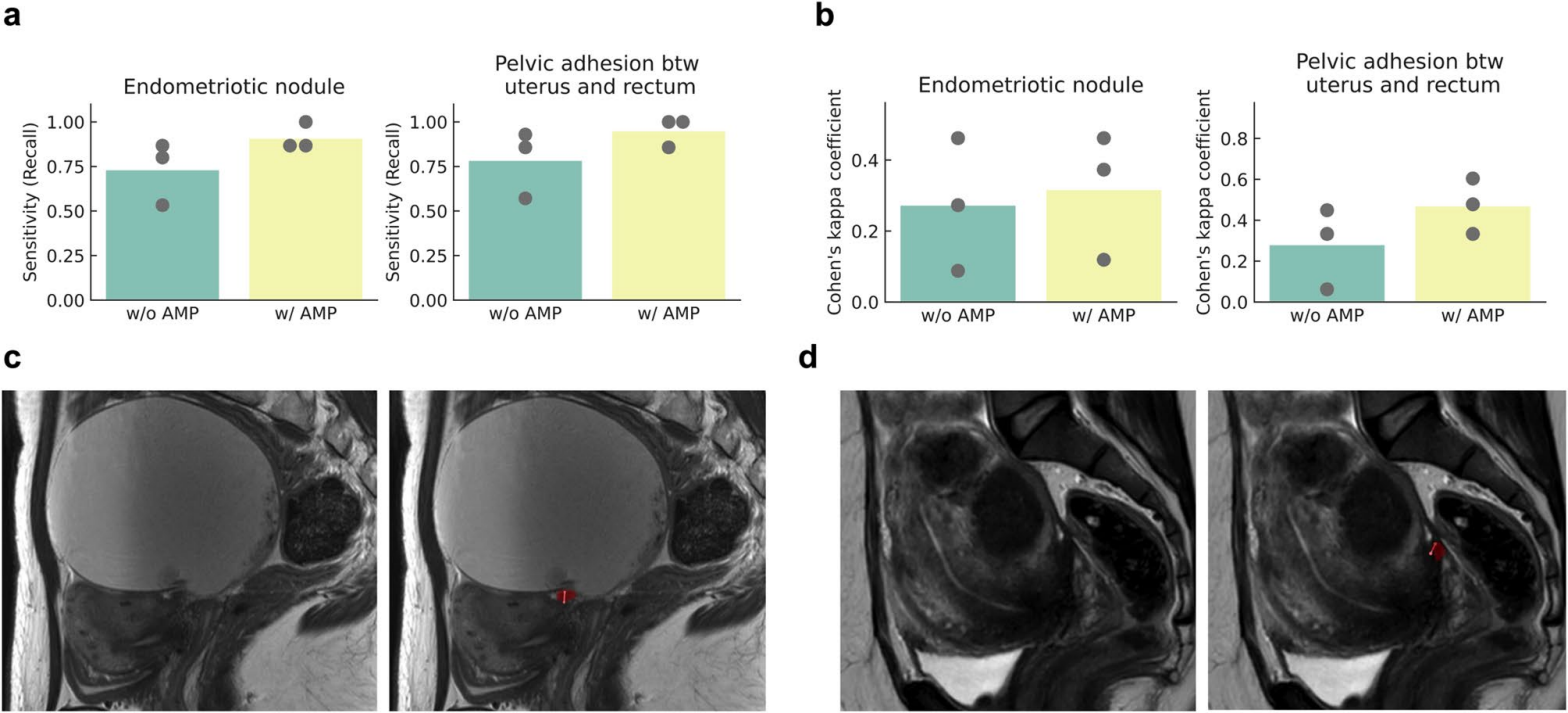
Assessment of endometriotic nodules and pelvic adhesions by radiologists. a, b The sensitivity and Cohen’s kappa coefficient of the research radiologists’ assessments with and without the assistance of AMP, regarding endometriotic nodules (plaques, left side) and pelvic adhesions between the uterus and rectum (right side). The points represent the scores for each research radiologist and the height of the bars represent their average. c, d Patient MR images where research radiologists initially failed to identify plaques without AMP. The left image in each set depicts the original patient MR images, while the right image exhibits the model’s prediction (indicated by a red segmentation mask) superimposed with one of the research radiologists’ measurements (in pink), which were made after consulting by the model’s prediction. In c, without AMP, all three research radiologists missed the plaque. With AMP, two out of the three identified the plaque as mild while the third identified none. In d, two research radiologists, initially missing the plaque without AMP, evaluated the plaque as mild and severe, respectively, after consulting the model predictions. The third radiologist consistently identified the plaque as severe with and without AMP.

### nnU-Net based model for plaque and OEC detection

For both the plaque and OEC model, we utilized the nnU-Net architecture^23,24^ (Fig. 1). On account of the inherent imbalance in the target voxel ratio, both the focal loss^29^ and Dice loss^30^ were used in a 1 : 1 ratio. The plaque segmentation model takes T2WI sagittal sequences as input, and outputs segmentation masks of the uterus, bladder, rectum, ovary, and plaque (Fig. 1a). The OEC segmentation model takes T1WI axial sequences as input, and outputs segmentation masks of the OEC (Fig. 1c).

To evaluate plaque location identification, we applied an object detection assessment approach. We encapsulated both the plaque segmentation mask and the ground truth plaque segmentation mask within bounding boxes. Sample images are shown in Fig. 2a, b. A prediction was classified as a true positive when the intersection over union (IoU) exceeded a set threshold of 0.01, accounting for plaque shape variability and the three-dimensional prediction space.

During preliminary explorations, the model could detect the majority of OECs, while it occasionally failed to identify the boundaries of the OECs. To address this issue, we incorporated a boundary loss alongside the focal and Dice loss for OEC segmentation^31^.

For both the plaque and OEC segmentation, we independently trained five models premised on varying random initializations, and the final segmentation predictions were acquired from the ensemble mean of these five models. Each model was trained across four NVIDIA A100 graphics processing units (GPUs) for a total of 511 epochs.

### Measurements for plaque depths and major and minor axes length of OEC

To assess the severity of the plaque, the depth from the posterior serosal surface of the uterus for each segmentation result was measured on the slice where it was largest. The measurement process for each slice involved the following steps: first, we identified the contact point between the uterus and the plaque by detecting the contour of the plaque within a predetermined distance from the boundary of the uterus. Next, we connected the two endpoints of this contact surface to form a line that is parallel to the posterior surface of the uterus. By adjusting the intercept of this line, we determined two straight lines that effectively enclose the plaque. The depth of the plaque was then calculated as the distance between these two bounding lines. We performed this measurement across all slices of the MR images, with the maximum depth observed among the slices reported as the depth of each plaque.

To measure the lengths of the major and minor axes of the OEC, we fitted the segmentation of each lesion on the MR image slices to an ellipse using the OpenCV library^32^. The major and minor axes for each slice were defined based on the fitted ellipse. We identified the slice containing the ellipse with the largest area and reported its dimensions as the major and minor axis lengths for each OEC. Additionally, to evaluate the change in volume of OEC before and after laparoscopic surgery, 54 image pairs from the 108 whole images evaluated for the OEC model were used to compare between model predictions and the ground truth by the proficient radiologists. The volume of each OEC was approximated by counting the total number of voxels within its segmentation mask.

### Radiomics-based model for detecting adhesions

The model that predicts the presence or absence of inter-organ adhesions in seven specific locations in the pelvic cavity was developed. It accounted for seven specific adhesion locations (unpublished, manuscript in preparation): (i) left ovary and rectum, (ii) right ovary and rectum, (iii) left and right ovary, (iv) uterus and left ovary, (v) uterus and right ovary, (vi) uterus and rectum, and (vii) uterus and bladder. Due to the high-dimensionality inherent in MR images and the limited availability of labels, we performed input dimension reduction by utilizing radiomics features^26,33^ rather than relying directly on the MR images. Radiomics technologies utilize either engineered hard-coded features or those derived through deep learning methods extracted from MR, positron emission tomography (PET), or CT images. Their efficacy in evaluating tumors has been demonstrated across a variety of tumor types, including brain, cervix, and lung cancers^34^.

Our approach involved the extraction of hard-coded shape-based features from organ segmentation predictions generated by the plaque segmentation model (Fig. 1a). For each MR image, we extracted the segmentation masks for the uterus, bladder, rectum, and ovary and calculated 14 unique shape features for each organ. These shape features comprised elongation, flatness, sphericity, major/minor axis length, the axis length in the direction of the largest principal component, the maximum diameter in the height/width/depth direction, 3D maximum diameter, mesh/voxel volume, surface area, and the surface and volume ratio. The resulting output is a 56-dimensional shape-based radiomics feature vector for every MR image. For each MR image and target organ, the presence or absence of adhesion was determined via consensus among three expert radiologists and human-in-the-loop by single expert radiologist. A LightGBM^25^ based model, using the 56–dimensional feature vectors as input, was utilized in predicting the binary adhesion labels (Fig. 1b).

### Preliminary investigation of clinical utility

A preliminary study was conducted to evaluate the clinical utility of AMP. We aimed to assess trends in the difference of reading accuracy across cohorts of reading with or without AMP. In this preliminary study, a total of 30 MR images selected from a dataset consisted of 333 MR images so that the distribution of severity for plaque and adhesion matched those of the entire dataset. Both over- and under-diagnosed cases (four images each) of the prediction results in plaque model were included to assess radiologists’ sensitivity to incorrect model predictions. This study employed a crossover design consisting of two parts (Part A and Part B) to alleviate potential biases. Cohort 1 in Part A (without AMP) and Cohort 2 in Part B (with AMP) consisted of the same 10 MR images with different numberings. Similarly, Cohort 2 in Part A (with AMP) and Cohort 1 in Part B (without AMP) utilized the same 10 MR images. A one-week interval was set between Part A and Part B for avoiding the memory biases. Cohort 3, used as a control group, contained the same MR images in both parts and the reliability of interval period was evaluated based on the results of this cohort. The interpretation of MR images was performed by three research radiologists. They had varying lengths of experience in gynecology imaging: Y.Hi. (1 year), Y.Ha. (5 years), and R.T. (15 years). The three research radiologists independently evaluated the presence of plaques and inter-organ adhesions in the pelvic cavity and were asked to measure the depth of plaques to categorize the plaque severity: none (zero mm), mild (under 5 mm), or severe (5 mm or more). Assessments in each cohort were required to be completed within one day whenever possible. The ground truth for the plaque and adhesion assessment was determined based on the consensus of three other expert radiologists according to the specific MR image reading protocol in the original study from which each data was generated.

## Results

### Efficacy of the nnU-Net based model for accurate plaque segmentation and localization

We assessed the in-domain performance of the nnU-Net based plaque segmentation model using a five–fold cross–validation methodology, ensuring MR images from the same patient remained within the same fold. The validation strategy employs 5–fold cross–validation. The entire dataset is divided into five non–overlapping stratified folds. The mean Dice Similarity Coefficient (DSC) values were 0.853 ± 0.008 for the uterus, 0.851 ± 0.008 for the bladder, 0.796 ± 0.007 for the rectum, 0.741 ± 0.016 for the ovary, and 0.293 ± 0.022 for plaques. In this study, the sigmoid colon was included in the “rectum” as a target area in some cases.

The out-of-domain performance of the plaque segmentation model was also evaluated by assessing the prediction accuracy using data from a facility not included in the training data. This was accomplished by omitting the MR images of twelve patients in the same facility from the training/validation data. The resultant DSC for the out-of-domain test set was 0.279.

While the nnU-Net-based model struggled with precise plaque volume prediction, it demonstrated high accuracy in identifying plaque location and thickness. The resulting mean average precision (mAP) was 0.858, indicating high accuracy in plaque location detection. With a specific confidence threshold, the model achieved a recall of 1.00 and a precision of 0.82 for plaque location prediction.

We measured the depth of the plaque segmentations (given the model plaque segmentation results) and compared these depths with grand truth measurements from experienced radiologists specialized in gynecological imaging (expert radiologist) (Figs. 2c, d). Pearson’s correlation coefficient between the model’s plaque depth measurements and those of expert radiologists was 0.52, suggesting the model’s credibility in providing initial plaque condition assessments (Fig. 2d).

### Robust OEC segmentation and volumetric analysis

The performance of the OEC model was gauged using identical data splits as employed in the plaque segmentation model. Furthermore, we incorporated a boundary loss in addition to focal and Dice loss.The resultant mean Dice score was 0.580 with boundary loss compared to 0.576 without it.

To assess clinical relevance, we compared the model’s predictions of major and minor axes, key metrics in OEC size evaluation, against ground truth segmentations (Fig. 3a). The results demonstrated strong concordance between predicted and ground truth measurements, with Pearson’s correlation coefficients of 0.97 and 0.98 for the major and minor axis, respectively (Fig. 3b, c).

To further scrutinize the OEC segmentation model’s effectiveness, we compared OEC volume changes pre- and post-surgery between model predictions and ground truths by the radiologists who have specialized experiences in gynecology imaging in a contract research organization (proficient radiologists) (MNES Inc., Hiroshima). The Pearson’s correlation coefficient calculation revealed a strong correlation (0.96; Fig. 3d) between the volume changes of OEC discerned by model prediction and those measured from proficient radiologist’s annotations.

### Efficacy of radiomics-based detection for inter-organ adhesions

Adhesions that may be associated with severe pelvic pain are frequently observed in DE. We therefore evaluated the ability of a radiomics-based model to predict the presence or absence of adhesions at specific locations in pelvic cavities. The results varied with the number of positive samples in the data, i.e. samples with one or more adhesions (Table 1). We found that adhesions related to the uterus generally demonstrated higher recall and precision compared to other areas. For adhesions related to the uterus other than the uterus and bladder, the model yielded recall and precision scores exceeding 0.6, which are comparable to the expert radiologist’s assessments (Table 1).

**Table 1:** Performance outcomes of the adhesion classification model in predicting the occurrence of organ adhesions.

Location	Recall	Precision	F1 score	Number of positive samples
Left ovary and rectum	0.70 (0.58–0.82)	0.40 (0.37–0.43)	0.48 (0.38–0.57)	126 (38%)
Right ovary and rectum	0.78 (0.69–0.87)	0.42 (0.35–0.50)	0.52 (0.42–0.61)	123 (37%)
Left and right ovaries	0.78 (0.55-1.00)	0.21 (0.18–0.25)	0.48 (0.44–0.52)	52 (16%)
Uterus and left ovary	0.85 (0.75–0.94)	0.77 (0.73–0.80)	0.58 (0.51–0.65)	242 (73%)
Uterus and right ovary	0.82 (0.72–0.91)	0.74 (0.67–0.81)	0.63 (0.57–0.68)	220 (66%)
Uterus and rectum	0.79 (0.73–0.85)	0.68 (0.63–0.73)	0.59 (0.52–0.65)	207 (62%)
Uterus and bladder	1.00 (1.00–1.00)	0.06 (0.02–0.10)	0.06 (0.02–0.09)	21 (6%)

The recall, precision, and F1 score metrics were computed based on a five-fold cross-validation of the entire dataset. ’Number of positive samples’ represents the number of patients who had organ adhesion at the specified location within the total dataset. Metrics achieving a score above 0.6 are highlighted in bold. Parentheses indicate 95% confidence intervals.

### Preliminary clinical utility study involving radiologists

We conducted a preliminary clinical utility study for AMP where three radiologists who have varying levels of experiences in gynecology imaging assessed endometriosis severity on 30 MR images, with or without the assistance of model predictions. Figure 4 represents the MR images interpretation results from research radiologists. We calculated recall (sensitivity) and Cohen’s kappa coefficient for the assessments both with and without AMP support (Fig. 4a, b and Supplemental Figs. 1 and 2). A trend toward improvement in the mean sensitivity and Cohen’s kappa coefficient was observed for plaques, as well as inter–organ adhesion including uterus and rectum, uterus and left ovary and uterus and right ovary (Table 2). The mean values of each plaque interpretation accuracy index increased from 0.73 to 0.91 and 0.27 to 0.32, respectively. In the 12 out of 20 cases where the model accurately diagnosed plaque severity, mean sensitivity and Cohen’s kappa coefficient showed a further increase from 0.67 to 0.93 and 0.22 to 0.65, respectively (data not shown). For inter-organ adhesions in pelvic cavities, the sensitivity increased 1.2 to 1.3–fold for the uterus and rectum, uterus and left ovary and uterus and right ovary, and the Cohen’s kappa coefficient showed 1.0 to 2.4–fold increase in the same location plus left ovary and rectum and right ovary and rectum. For two locations (left and right ovary, uterus and bladder), we could not calculate the accuracy due to the lack of positive samples within the images used in the preliminary clinical utility study.

**Table 2:** The mean sensitivity (recall) and cohen’s kappa score of the three research radiologists’ assessment with and without AMP in plaque and adhesion detection in the preliminary clinical utility study.

Location	Sensitivity		Cohen’s kappa coefficient	
	w/o AMP	w/ AMP	w/o AMP	w/ AMP
Endometriotic nodule (plaque)	0.73 (0.29, 1.00)	0.91 (0.72, 1.00)	0.27 (-0.19, 0.74)	0.32 (-0.12, 0.76)
Uterus – Rectum	0.79 (0.32, 1.00)	0.95 (0.75, 1.00)	0.28 (-0.21, 0.78)	0.47 (0.14, 0.81)
Left ovary – Rectum	0.40 (-0.59, 1.00)	0.27 (-0.02, 0.55)	0.07 (-0.58, 0.73)	0.16 (-0.11, 0.43)
Right ovary – Rectum	0.80 (0.30, 1.00)	0.73 (0.16, 1.00)	0.49 (-0.06, 1.00)	0.50 (0.19, 0.81)
Uterus – Left ovary	0.67 (-0.08, 1.00)	0.89 (0.77, 1.00)	0.18 (-0.05, 0.41)	0.34 (0.11, 0.57)
Uterus – Right ovary	0.59 (-0.31, 1.00)	0.72 (0.10, 1.00)	0.14 (0.08, 0.19)	0.34 (-0.26, 0.94)
Left ovary – Right ovary	–	–	–	–
Uterus – Bladder	–	–	–	–

Plaque assessment was conducted using binary (recall) and ternary (Cohen’s kappa) classifications. Adhesion assessment was conducted using binary classification for both recall and Cohen’s kappa. Results for adhesions between the left ovary–right ovary, and uerus–bladder could not be calculated due to the lack of positive samples within the images used in the preliminary clinical utility. Parentheses indicate 95% confidence intervals.

Calculating the average Cohen’s kappa for plaque detection among different readers, we found that it was 0.356 (–0.196, 0.907) without AMP, whereas with the addition of AMP, it increased to 0.562 (0.418, 0.707), indicating improved agreement. This trend was similarly observed for adhesion detection in nearly all regions.

Identifying plaques is sometimes challenging, even for expert radiologists. Figure  4c, d represent two cases where research radiologists failed to identify plaques without model assistance. In Fig. 4c, all three of them missed the plaque without AMP, while two classified it as mild after referring to the AMP prediction. In Fig. 4d, two out of three research radiologists, who initially missed the plaque without AMP, correctly identified it as mild and severe, respectively, after consulting with AMP.

Model assistance also appeared to positively impact the detection of pelvic inter-organ adhesions, particularly those to uterus (Table 2). Specifically, Fig. 4a, b indicates that incorrect interpretations regarding the presence of adhesions between the uterus and rectum were rectified in eight out of ten cases initially missed by the radiologists without AMP assistance. In addition, AMP support was less effective for adhesions between the left ovary and rectum and between the right ovary and rectum shown in Table 2.

## Discussion

### Study objective and summary of findings

As stated in the introduction, the primary endpoint of this study was to develop AMP and to demonstrate its potential to improve radiologists’ reading sensitivity. Considering that this study was an exploratory phase, we did not define a fixed numerical success criterion but set a qualitative one that AMP would improve radiologists’ reading accuracy. In a preliminary small-scale study, it was suggested that AMP may contribute to improved reading sensitivity in specific diagnostic domains. Subjective analysis further indicated that AMP could be a useful tool for lesion detection and workload reduction.

AMP’s performance is supported by several technical innovations. The use of 3D multi-slice MRI processing enabled volumetric feature extraction of pelvic anatomy, while ensemble learning improved robustness and generalization. Radiomics-based features, particularly shape descriptors from organ segmentations, allowed the adhesion classification model to detect subtle morphological changes. This hybrid approach combines deep learning with domain-specific anatomical knowledge.

While AI has advanced in static lesion detection (e.g., brain and lung), pelvic imaging with the presence of peristaltic organs like the bowel and ureters^35–42^ remains challenging due to organ motion. Reviews on AI studies in endometriosis imaging are limited^28–34^. In a comprehensive review published by Avery et al.^35^, they found only two MRI-based AI studies. Jiang et al.^42^ noted that manual diagnosis remains necessary for ovarian endometriosis, and Butler et al. focused on self-supervised pre-training and the pouch of Douglas^22^.

By incorporating segmentation of surrounding pelvic organs (uterus, bladder, rectum, ovaries), AMP enhanced the contextual understanding of lesion location and morphology. This is especially effective in the pelvis, where organ motion and anatomical variability complicate interpretation. AMP’s ability to detect plaques and adhesions in such environments represents a meaningful advancement over conventional rule-based or purely visual interpretation methods. The plaque segmentation model, despite its modest DSC, achieved high mean average precision (mAP 0.858), which is valuable given the subtle and complex nature of DE lesions. In a preliminary reader study, AMP improved plaque detection sensitivity from 0.73 to 0.91 (Table 2), suggesting its potential to enhance diagnostic accuracy and reduce inter-reader variability.

While the use of an IoU threshold of 0.01 for plaque localization may raise concerns regarding potential inflation of performance metrics, and more standard thresholds such as IoU ≥ 0.25 or ≥ 0.5 are generally recommended in lesion detection tasks, the threshold of IoU ≥ 0.01 was considered appropriate for the following reasons. First, the standard threshold adopted in a nodule detection service approved by the Pharmaceuticals and Medical Devices Agency of Japan (PMDA) were referred, where IoU ≥ 0.01 was accepted as a valid criterion^43^. The validity of the performance accuracy is further supported by the adoption of assessment criteria derived from medical device programs whose clinical utility has been officially recognized. Moreover, an IoU ≥ 0.01 has been widely used in previous peer-reviewed studies^44–46^ across various medical imaging tasks (multiple sclerosis lesions, prostate lesions, and intracranial aneurysms) supporting its academic validity.

The OEC segmentation model achieved a mean DSC of 0.580 with boundary loss and demonstrated strong agreement with expert radiologists in measuring lesion dimensions and volume changes before and after surgery. The Pearson’s correlation coefficient for volume change was 0.96 (Fig. 3d), indicating that AMP can provide reliable metrics for treatment planning and monitoring. The radiomics-based adhesion classification model achieved recall and precision scores exceeding 0.65 for uterine-related adhesions^47^ (Table 1), which are clinically relevant for surgical decision-making.

Improved diagnostic accuracy has broad clinical impact. As Bruyere et al.^14^ reported, radiologist experience affects performance. AMP showed consistent utility across experience levels, improving sensitivity and inter-reader agreement (Fig. 4a and b). In some cases (Fig. 4c and d), AMP helped identify previously missed lesions, supporting less experienced readers and reducing variability. Details of the study design in this preliminary study, including image selection and the washout period, are described in the “Materials and methods” section. This is particularly valuable in settings with limited access to specialized radiologists, highlighting AMP’s potential to democratize expertise and improve care quality.

Regarding a subjective analysis of the clinical needs of AMP for plaques, adhesions, and OECs, AMP was recognized with potential clinical utility in lesion detection and workload reduction, and there is also growing interest in its applicability to other disease areas.

### Limitations

This study has several limitations. First, the Dice Similarity Coefficient (DSC) for plaque segmentation was relatively low (in–domain 0.293, out–of–domain 0.279), reflecting the technical difficulty in delineating small (5–30 mm), irregular lesions with indistinct boundaries. This low DSC can be attributed to the inherent challenges in segmenting plaques with subtle and complex features, which are difficult to annotate even for experienced radiologists. This annotation variability likely impacted the quality of the training data, thereby limiting the model’s segmentation accuracy.　Bruyere C et al.^14^ reported a significant gap in diagnostic accuracy among radiologists for the posterior pelvic compartment. While experts achieved an AUC of 0.92, those with ≤ 2 years of experience showed lower performance (AUC 0.65–0.81, *p* < 0.001). This highlights the steep learning curve in interpreting complex pelvic MRI findings, especially for DE. Therefore, these characteristics make accurate annotation challenging even for expert radiologists, and such variability in ground truth may have influenced the model’s training and evaluation. Although the model achieved a high mAP of 0.858 for plaque localization (Fig. 2b), the low DSC indicates that volumetric segmentation remains difficult, particularly for lesions with subtle margins.

Second, the dataset used in this study was relatively limited in size and diversity, comprising 333 MR images from 277 patients. While this number is substantial for clinical research, it remains small for training deep learning models such as nnU-Net, which typically require thousands of cases to achieve robust performance. Moreover, DE is highly heterogeneous in terms of lesion location, size, and signal characteristics. Such variability is difficult to capture comprehensively in a dataset of this scale. The imbalance across lesion types—for example, only 21 cases of uterus–bladder adhesions—further explains the model’s uneven performance, with higher accuracy at frequent sites and reduced performance at rare ones. These limitations underscore the need for larger, more balanced datasets to improve model generalizability and robustness. Although we prioritized feasibility by using a single MR imaging sequence per model, processing it as a 3D volume using a 3D convolution-based architecture due to constraints in data availability, annotation workload, and computational resources, a multi-planar or fully 3D approach that integrates multiple sequences could potentially better capture anatomical variability and enhance model robustness in future studies.

Third, the scope of validation for lesion and adhesion assessment was limited. For plaques, segmentation was restricted to the posterior uterine surface, excluding other common and clinically important sites such as the uterosacral ligaments and rectovaginal septum. For adhesions, ground truth labels were determined solely based on radiological interpretation without surgical confirmation, which may compromise the reliability of the reference standard. These limitations narrow the applicability of the study findings and may reduce the generalizability of AMP to broader clinical scenarios. In addition, each participating center contributed a relatively small number of cases, which may have limited the diversity of scanner types and imaging protocols represented in the dataset. This raises concerns about the generalizability of AMP to external, real-world clinical settings. Moreover, as already mentioned, the definitive diagnosis and classification of endometriosis require laparoscopic and histopathological confirmation. Since this study relied solely on MRI without surgical validation, there is an inherent risk of diagnostic bias. In particular, the presence of adhesions cannot be definitively confirmed without laparoscopy, which limits the reliability of the ground truth labels used for training and evaluation. This limitation should be considered when interpreting the model’s performance.

Finally, the study employed a retrospective design, and the clinical utility assessment was based on a small-scale, preliminary reader study. Although the results were encouraging, the lack of prospective validation and limited comparison with laparoscopic findings—the current gold standard for DE diagnosis—restricts the strength of the conclusions. Further validation in larger, prospective, and multi-center studies is necessary to confirm the clinical effectiveness of AMP.

### Future work

As previously mentioned, several research challenges must be addressed to enhance the clinical applicability and robustness of AMP. For successful implementation in clinical practice, integration with existing healthcare information systems is essential. Embedding AMP into Picture Archiving and Communication Systems (PACS) and electronic health records would allow radiologists to access its diagnostic support seamlessly within their daily diagnostic workflow. In the future, in addition to further pursuit of robust performance that is not affected by lesion localization, the incorporation of multimodal approaches—combining MRI with ultrasound, clinical history, or lab data— and the development of predictive models using longitudinal imaging may support treatment monitoring and personalized care.

## Conclusion

AMP developed in this study demonstrated promising utility for the non-invasive diagnosis of DE and OECs. By integrating three complementary models—plaque segmentation, adhesion classification, and OEC quantification—AMP offers a comprehensive diagnostic support system for DE imaging.

Collectively, these findings support the potential of AMP to improve diagnostic performance, reduce diagnostic delays, and support radiologists in identifying subtle DE lesions and complex adhesion patterns. This study should be regarded as a proof-of-concept that provides preliminary evidence of AMP’s utility, rather than a clinically validated tool. Limitations such as the relatively small and imbalanced dataset, lack of surgical gold-standard validation, use of non-standard evaluation metrics, and the small-scale reader study restrict the generalizability and strength of the conclusions. Further large-scale, prospective validation studies are necessary to establish the clinical effectiveness and real-world applicability of AMP.

Beyond immediate clinical utility, integrating AI into healthcare systems contributes to broader goals such as advancing women’s health, reducing disparities, and promoting sustainability. With continued refinement and validation, AMP may become a transformative tool in reproductive health.

## Supplementary Information

Below is the link to the electronic supplementary material.

Supplementary Material 1

## Data Availability

The datasets generated during or analyzed during the current study are available from the corresponding author upon reasonable request.
