# Supplementary material for: Development of an AI-based magnetic resonance imaging reading support program (AMP) for deep endometriosis diagnosis
Source: Sci Rep. 2025 Dec 8;16:790. doi: 10.1038/s41598-025-30277-x (PMC12780234; doi:10.1038/s41598-025-30277-x)

Supplemental Figure 1. Sensitivity of the research radiologists' assessment with and without AMP in plaque and adhesion detection in the preliminary clinical utility study

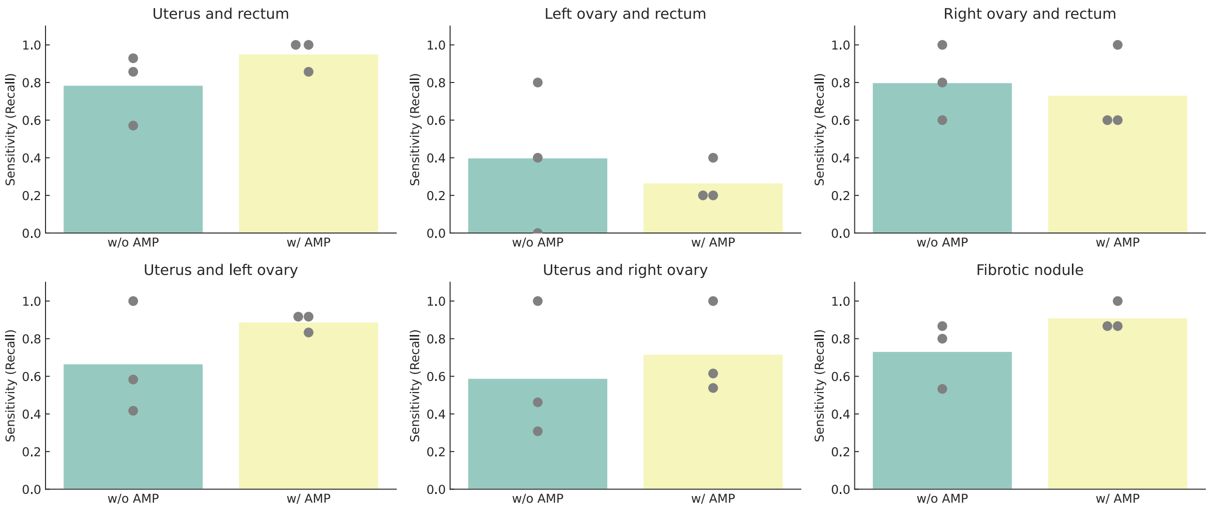

Supplemental Figure 2. The Cohen's kappa coefficient of the research radiologists' assessment with and without AMP in plaque and adhesion detection

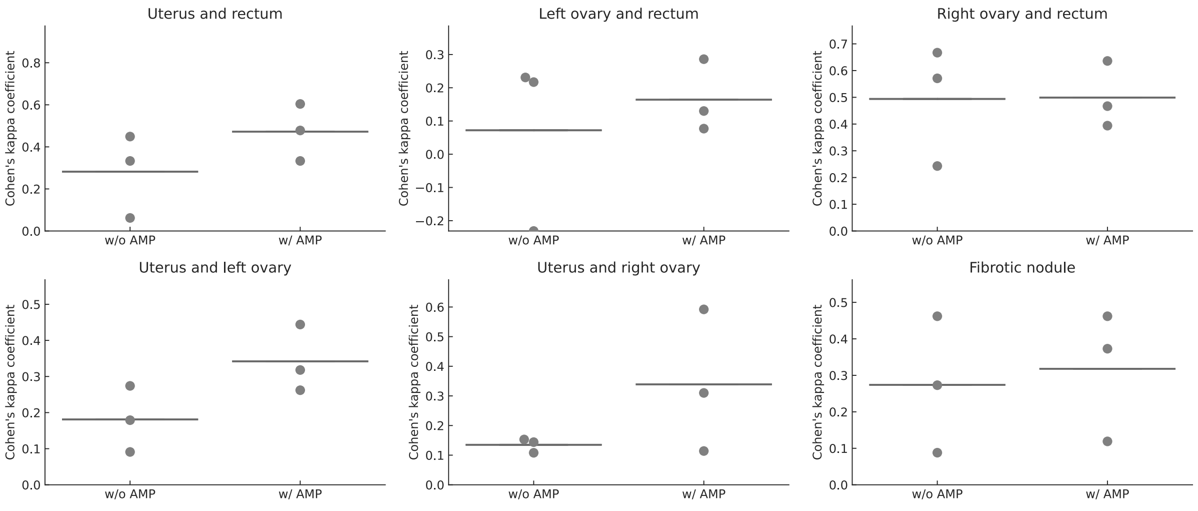

Supplement: Supplementary file 1 — Supplementary Material 1 [file 41598_2025_30277_MOESM1_ESM.pdf]
